# Reply to Comment—Osteonecrosis of the Jaw in Myeloma Patients Receiving Denosumab or Zoledronic Acid. Comment on Pivotal Trial by Raje et al. Published in Lancet Oncology

**DOI:** 10.3390/dj7020054

**Published:** 2019-05-16

**Authors:** Noopur Raje, Evangelos Terpos, Danielle D. Jandial

**Affiliations:** 1 Center for Multiple Myeloma, Massachusetts General Hospital Cancer Center, Boston, MA 02114, USA; 2 School of Medicine, National and Kapodistrian University of Athens, Alexandra General Hospital, 11528 Athens, Greece; eterpos@hotmail.com; 3 Amgen Inc., Thousand Oaks, CA 91320, USA; djandial@amgen.com

We read the comment by Fusco et al. [1] in response to our recent article in *Lancet Oncology* titled “Denosumab Versus Zoledronic Acid in Bone Disease Treatment of Newly Diagnosed Multiple Myeloma: An International, Double-Blind, Double-Dummy, Randomised, Controlled, Phase 3 Study” [2], and want to thank the authors for their interest and review of our study report. We would like to respond to their comment as follows.

## 1. There Was Higher Rate of Osteonecrosis of the Jaw (ONJ) Reported in the Multiple Myeloma Study Compared with Other Pivotal Trials (Despite Similar Drug Exposure)

The incidences of “adjudicated” ONJ reported for denosumab (4.1%) and zoledronic acid (2.8%) in our study are higher than those reported in other pivotal trials of denosumab versus zoledronic acid in patients with metastatic solid tumor malignancies [3,4,5,6]. For example, results from an integrated dataset from three pivotal denosumab registrational studies in 5723 patients with bone metastases and solid tumors (97%) or multiple myeloma (3%) reported lower incidences of adjudicated ONJ of 1.8% in the denosumab group and 1.3% in the zoledronic acid group [6] than that observed in our study of patients with multiple myeloma. This difference in ONJ rates reported with skeletal-related events secondary to hematologic malignancies involving bone versus solid tumors was higher in both treatment arms, consistent with previously reported bisphosphonate trials [7,8,9,10,11,12,13,14,15]. There are several factors that may contribute to this observation. First, the totality of the data suggests that myeloma patients may be at a higher inherent risk for ONJ development, potentially related to the dysregulated bone microenvironment secondary to the disease state or to the use of concurrent medications for anti-myeloma therapy, in particular high-dose corticosteroids, which have been associated with ONJ [11,16]. ONJ risk is significantly increased with oral infection [17], which may also be an important contributor. Second, the incidence of ONJ increases with antiresorptive therapy duration of exposure and cumulative dose [9,18,19,20]. Consistent with this observation, median exposure to denosumab and zoledronic acid was longer in our study (15.8 and 14.8 months, respectively) [2] than that reported in the integrated dataset from pivotal denosumab registrational studies in solid tumor patients (12.0 and 11.1 months, respectively) [6,21]. The data presented in Fusco et al. [1] compare cumulative incidence of ONJ across the various trials. Because cumulative incidence depends on the patient characteristics, treatment characteristics, and time period of follow-up, it is difficult to compare or summarize the cumulative incidences in a single summary risk estimate across studies. Therefore exposure-adjusted ONJ incidences, adjusted for patient years of follow-up to reflect different lengths of time on study, are provided to further characterize differences. Exposure-adjusted ONJ incidence rates in denosumab patients in the integrated dataset from pivotal denosumab registrational studies in patients with breast or prostate cancers were 1.1 per 100 patient-years in the first year of treatment, 3.7 in the second year, and 4.6 per year thereafter; median time to ONJ was 20.6 (range 4–53) months [20,22]. The corresponding exposure-adjusted ONJ incidence rates for denosumab in our study were 2.0 per 100 patient-years in the first year, 5.0 in the second year, and 4.5 per year thereafter; median time to ONJ was 18.7 (range 1–44) months [20,22]. Finally, other contributing factors to a higher ONJ incidence might include increased event recognition by investigators or a potential impact of the 2014 revision to the American Association of Oral Maxillofacial Surgeons (AAOMS) criteria expanding ONJ diagnostic criteria to include fistula.

## 2. How Many Patients Received Dental Procedures Overall, That Is, the Global Treated Population, and Their Reasons?

At enrollment, non-healed dental or oral surgery was a key exclusion criterion (oral examinations performed every 6 months) and a protocol amendment mandated antiresorptive medication discontinuation 30 days before an elective invasive oral or dental procedure and until complete healing occurred. Nonetheless, invasive dental procedures were reported as a main risk factor in more than one half of patients (54% of patients in both groups) with adjudicated ONJ, suggesting that emergent dental procedures continued to occur while on therapy. A prior prospective trial on ONJ prevention in patients with prostate cancer treated with zoledronic acid demonstrated that more frequent surveillance and preventive dentistry decreases ONJ incidence, which may in part be due to managing oral risk factors before more invasive dental procedures are undertaken [23].

## 3. ONJ Cases May Have Been Overlooked Due to a Change in ONJ Criteria during the Trial; How Did This Change in Criteria Influence the Results?

The definition of ONJ according to the AAOMS was revised slightly during the time-period of the trial to include cases without bone exposure but only if bone can be probed through a fistula [18]; therefore, the ONJ definition used in the current trial was also amended. This may have been a factor in the higher event rates observed in this trial compared with the older skeletal-related events (SRE) trials (all pre-2014, so the previous AAOMS guidelines were used). The blinded adjudicators in this study followed standard dental consensus guidelines, rather than the definition presented in the protocol. Additionally, because they were independent reviewers, the protocol was not shared with them.

## 4. What Are the Numbers of “Potential” ONJ Cases Registered by Investigators, and Defined by the Presence of Clinical Signs and Symptoms Suggestive of ONJ, in Both Treatment Arms? Are These Rates Comparable to those from Another Solid Tumor Study (Saad et al., 2012) That Reported That Only One-Third of Potential ONJ Cases Were Adjudicated?

**Figure 1:**
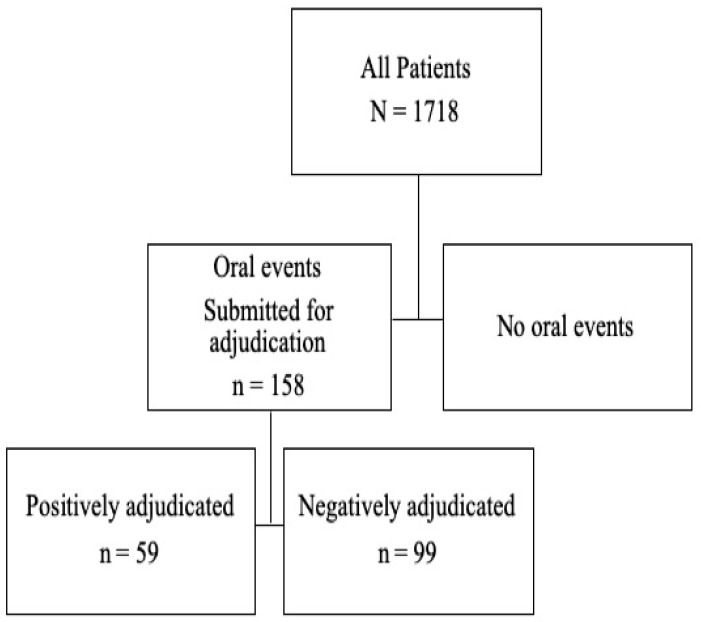
Summary of adverse oral events submitted for adjudication.

We believe that the information reported in the Saad et al. 2012 study may have been misinterpreted by Fusco et al. [1]. In that study, all of the oral adverse events (AEs) were referred to the independent ONJ adjudication committee and, of those, one third of the cases evaluated were found to be positively-adjudicated ONJ. The adjudication process used in both studies drew from a broad and robust list of AE preferred terms (approximately 40 in total) that were identified using the Medical Dictionary for Regulatory Activities (MedRA) and clinical review of all adverse oral events. Any reported AE that matched a term from the compiled list was pulled for adjudication by two independent blinded dental experts, and both reviewers had to agree for the event to be considered positively adjudicated. Given the breadth of the list consulted, it is not surprising that only a minority (about one third in both studies) actually met the AAOMS criteria for ONJ. In this study, the number of potential oral events meeting criteria for adjudication was 158 events from 1718 (9.2%) patients compared with 287 events from 5723 (5.0%) patients in the Saad et al., 2012 study. Of the 158 events submitted for adjudication in our study, 59 patients (24 and 35 patients in the zoledronic acid and denosumab groups, respectively) were confirmed as having positively-adjudicated ONJ, representing 3.4% (59/1718) of the overall patient population (see Figure 1), compared with 1.6% (89/5723) of the overall patient population in the Saad et al. study [6].

## 5. Long-Term ONJ Estimates from the Multiple Myeloma Trial Are Awaited with Interest

There is no planned follow-up analysis according to treatment arm for the patients with multiple myeloma in this study beyond what has been reported in the Raje et al. publication [2]; however, at the end of the double-blind extension period, patients were offered the opportunity to continue denosumab therapy for up to an additional two years in an open-label extension (OLE) study. At the conclusion of this OLE study, an analysis of safety endpoints will be performed.

## 6. Reports on the Cost-Effectiveness of Denosumab Versus Zoledronic Acid in Myeloma Patients Are Needed

Recent data from a cost-effectiveness analysis of denosumab versus zoledronic acid, integrating data from the Raje et al. study [2] indicated that in the United States, from a societal perspective, the use of denosumab instead of zoledronic acid resulted in a cost per quality-adjusted life year gained of $107,939 USD, and a net monetary benefit difference of $10,259 USD in favor of denosumab, owing to its combined impact on reducing skeletal-related events (SREs), potential to improve progression-free survival, and its lack of impact on renal function [24]. The model used in the analysis included utility decrements for SREs, mode of drug administration, serious adverse events (including ONJ and renal toxicity) and disease progression. Overall, these results suggest that denosumab is a cost-effective option compared with zoledronic acid for the prevention of SREs in multiple myeloma.

## 7. Summary

We agree with the authors of the comment that more information is needed regarding ONJ risk in patients receiving antiresorptive agents. There are several factors (e.g., duration of exposure and patient population) that may have contributed to the higher incidence of ONJ in both treatment arms observed in our analysis. However, until more data are available, and as noted in the prescribing information for denosumab (XGEVA^®^) [20], patients should undergo an oral examination before starting treatment. Once receiving treatment, patients need to be monitored for ONJ symptoms and should avoid invasive dental procedures. We have addressed as many of the concerns that were raised by Fusco et al. [1] as is possible, and again thank them for their comments and observations on our study results.
